# Outcomes With Left Ventricular Assist Device in End-Stage Renal Disease: A Systematic Review

**DOI:** 10.7759/cureus.24227

**Published:** 2022-04-18

**Authors:** Sofia Lakhdar, Mahmoud Nassar, Chandan Buttar, Laura M Guzman Perez, Shahzad Akbar, Anoosh Zafar, Most Munira

**Affiliations:** 1 Internal Medicine, Queens Hospital Center, New York City, USA; 2 Internal Medicine, Icahn School of Medicine at Mount Sinai, New York City Health and Hospitals/Queens, New York City, USA; 3 Internal Medicine, Icahn School of Medicine at Mount Sinai, Queens Hospital Center, New York City, USA; 4 Internal Medicine, Kettering Medical Center, Dayton, USA; 5 Medicine, Rawal Institute of Health Sciences, Islamabad, PAK; 6 Cardilogy/Medicine, Weill Cornell Medicine, New York City, USA; 7 Cardiology, Queens Hospital Center, New York City, USA

**Keywords:** heart assist devices, bridge to transplant, destination therapy, advanced heart failure, hemodialysis, chronic kidney disease (ckd), end-stage renal dysfunction, ventricular assist device

## Abstract

Renal dysfunction is a common comorbidity in patients with advanced heart failure who may benefit from mechanical circulatory support (MCS). Unfortunately, renal function may result after left ventricular assist device (LVAD) implantation. The purpose of this study is to examine the outcomes of advanced heart failure patients with end-stage renal disease (ESRD) requiring mechanical circulatory support as a bridge to transplant (BTT) or destination therapy (DT). We searched Medline, Embase, and Cochrane in September 2021. The following keywords were used: left ventricular assist device or LVAD and end-stage renal disease or ESRD. Our study included case reports, case series, descriptive studies, and randomized control trials. Review articles, guidelines, systematic reviews, and meta-analyses were excluded. We also excluded pediatric cases. We identified 278 articles; 92 were duplicated, 186 articles entered the screening phase, and 133 articles were excluded by title and abstract. After the full-text screening, 40 articles were excluded. This systematic review included 13 articles. Among the contraindications to LVAD implantation, a general contraindication is for patients found to have stage 4 chronic kidney disease (CKD) (estimated glomerular filtration rate (eGFR): <30 mL/minute/1.73 m^2^), while those on dialysis are an absolute contraindication LVAD implantation. Despite the limited data and publications on LVADs in patients with ESRD, LVAD implantation as a bridge to transplantation or destination therapy may be considered in selected patients without increasing morbidity and mortality. Therefore, shared decision-making around the treatment of advanced heart failure with these patients and the care team is essential.

## Introduction and background

Heart transplantation remains the standard of care for patients with end-stage heart failure; however, there remains a discrepancy between available donor hearts and recipients. Mechanical circulatory support (MCS), specifically left ventricular assist devices (LVADs), is widely used as destination therapy (DT) or as a bridge to transplant (BTT) in patients with advanced heart failure awaiting transplantation [1]. Several factors play a role in evaluating patients who will benefit from heart transplantation. Patients with chronic kidney disease (CKD) are usually at high risk of morbidity and mortality [1]. Renal dysfunction may range from early stages to end-stage renal disease (ESRD). It has been reported that renal dysfunction is associated with a reduced survival rate after LVAD implantation, especially in patients with ESRD at the time of implantation. This review addresses the outcomes in patients undergoing LVAD implantation with ESRD.

## Review

Methods

We utilized the Preferred Reporting Items for Systematic Reviews and Meta-Analyses (PRISMA) framework to perform our systematic review [2].

Search Strategy and Selection Criteria

We searched the following databases in September 2021: Medline, Embase, and Cochrane. The Medical Subject Headings (MeSH) terms included “left ventricular assist device,” “LVAD,” “end-stage renal disease,” and “ESRD.” We included case reports, case series, descriptive studies, and randomized control trials. Studies published in the English language were included. We have excluded review articles, guidelines, systematic reviews, and meta-analyses. We also excluded pediatric cases. All of the included articles were published in peer-reviewed journals.

Data Abstraction

The identified articles have been imported to the Covidence website for screening by two independent coauthors (SL and CB). In addition, a spreadsheet containing data was compiled by one author (CB) and verified by another author (NM). These articles were all published in peer-reviewed journals. Statistical analysis was performed using SPSS version 27 (IBM Corporation, Armonk, NY, USA). A descriptive analysis was performed to describe the results.

Results

**Figure 1:**
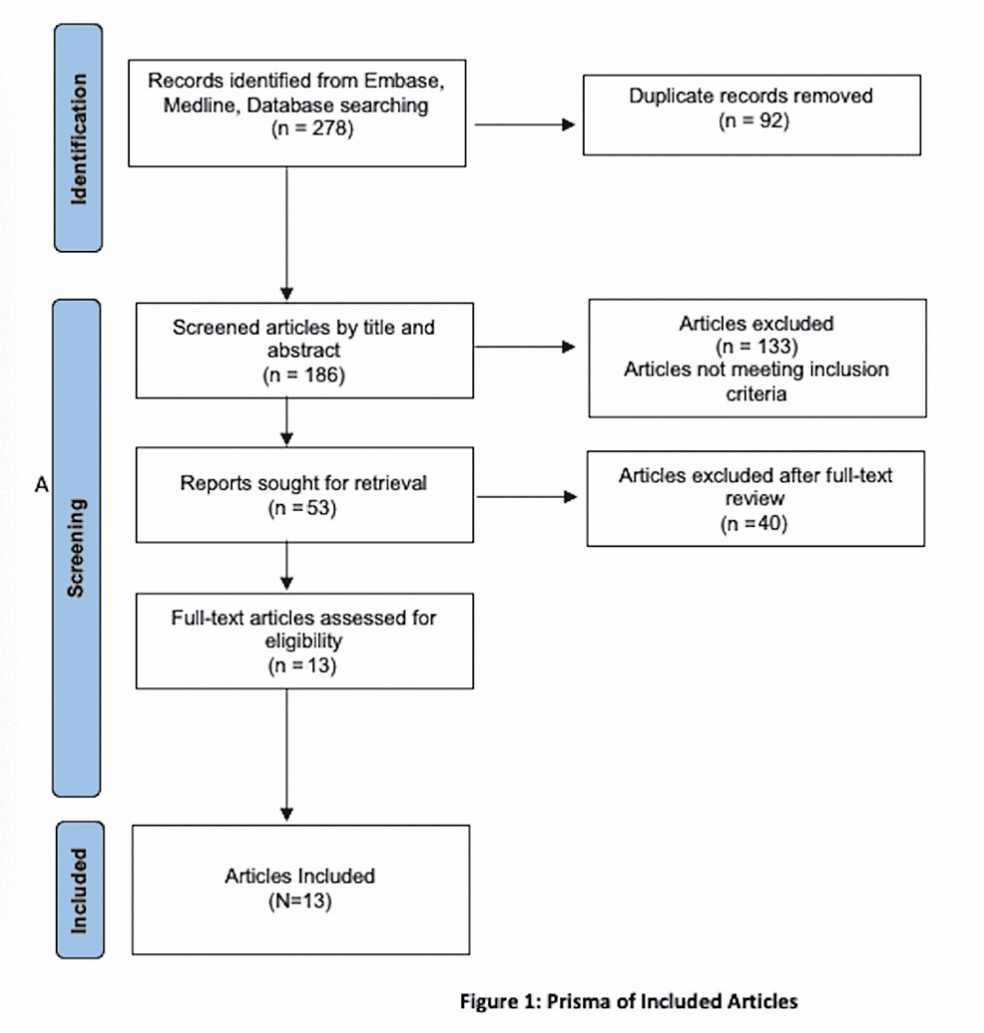
PRISMA flow diagram of the study screening process PRISMA: Preferred Reporting Items for Systematic Reviews and Meta-Analyses

We identified 278 articles; 92 were found to be duplicate records, 186 articles entered the screening phase, and 133 articles were excluded by title and abstract during the screening. Forty articles were excluded after full-text screening (Figure 1). This systematic review included 13 articles: six case reports, one case series, four retrospective descriptive studies, and two prospective studies. Table 1 evaluates the studies included and the clinical outcomes of patients with ESRD prior to LVAD placement. The baseline study characteristics are found in Table 2. Six case reports were included in this study and described six patients with ESRD, of which five cases had ESRD prior to undergoing LVAD placement. We included one case series of 11 patients, of which patients initially presented with CKD prior to LVAD placement; however, two patients were lost to follow-up, two patients died post-procedure, and three patients recovered renal function. One patient underwent a kidney transplant, while two patients were continued on both hemodialysis (HD) and LVAD support. A retrospective study described 127 patients (81.9%) with and 95 (36.4%) without ESRD who died. More than half of the patients with ESRD (80 (51.6%)) compared with 11 (4%) of those without ESRD died during the index hospitalization. However, neither highlighted that this risk is decreased in recent years in those with and without ESRD and becoming almost similar perhaps due to improved technology and experience. The ESRD group has similar in-hospital mortality as the non-ESRD group.

**Table 1:** Outcomes of patients with ESRD on LVAD

Number	First author	Outcome
1	Bansal (2018) [3]	During a median follow-up of 762 days (IQR, 92-3,850 days), 127 patients (81.9%) with and 95 (36.4%) without ESRD died. More than half of the patients with ESRD (80 (51.6%)) compared with 11 (4%) of those without ESRD died during the index hospitalization. The median time to death was 16 days (IQR, 2-447 days) for patients with ESRD compared with 2,125 days (IQR, 565-3,850 days) for those without ESRD. With adjustment for demographics, comorbidity, and time period, patients with ESRD had a markedly increased adjusted risk of death (hazard ratio, 36.3; 95% CI, 15.6-84.5), especially in the first 60 days after LVAD placement.
2	Walther (2018) [4]	Mortality during the implantation hospitalization was 40.6%. Within one year of implantation, 61.5% of people had died. On multivariable analysis, males had half the mortality risk of females. Lower mortality risk was also seen with VAD implantation in a primary setting and with a more recent year of implantation, but these results did not reach statistical significance.
3	Agarwal (2019) [5]	Intermittent hemodialysis (IHD) was tolerated well. CO increases significantly after ultrafiltration despite a decrease in mean Doppler pressure.
4	Bavishi (2018) [6]	Out of 29,247 patients, the overall readmission among ESRD patients was 3.2% (935); patients with diabetes (34.9%) and hypertension (64%) were found to have higher overall readmissions. Out of 4,535 patients, readmissions within 30 days included 4.9% of patients with ESRD. Out of an overall 24,712 who did not have readmission, 2.9% of ESRD patients did not have any readmissions. This study reviewed the baseline characteristics of 29,247 patients and their 30-day readmission. The major etiologies of 30-day readmission were congestive heart failure CHF (systolic or diastolic), with an incidence of 22%; stable CAD, 11.1%; and sepsis/septic shock, 4.6%. AKI was found in 2.6% of patients.
5	Demirozu (2011) [7]	Fifteen who had creatinine greater than two or 1.5 times pretransplant were started on renal replacement therapy (RRT) plus HD, and five became ESRD, requiring long-term dialysis. People who required RRT and remained stable on LVAD had renal function improvement within two months compared to prior LVAD.
6	El Sayed Ahmed (2014) [8]	The patient received a heart and kidney transplant and was discharged with good kidney and heart function on postoperative day 18.
7	Franz (2020) [9]	Eleven patients were included in the study; two patients were lost to follow-up. Out of nine patients, three recovered kidney function, one recovered kidney and heart function, one received a heart and kidney transplant, two continued with hemodialysis on LVAD support, and two died; patients on LVAD can tolerate hemodialysis and survive with outpatient hemodialysis centers and LVAD treatment teams working together.
8	Gilligan (2020) [10]	The patient died after three months but tolerated the peritoneal dialysis for these three months with conversion of PleurX catheter to a peritoneal dialysis catheter; although in the past he had a poor tolerance of hemodialysis, ascites and LVAD are both a contraindication to peritoneal dialysis.
9	Hankinson (2019) [11]	LVAD requires warfarin, which can increase calciphylaxis, especially in patients with sarcoidosis, ESRD on HD, and hypercalcemia, and should be diagnosed early; the patient died from other causes (hemorrhagic shock).
10	Ignaszewski (2017) [12]	Pannus formation is a concern in long-term LVAD therapy; the patient died five years posttransplantation and tolerated peritoneal dialysis during that time.
11	Shah (2017) [13]	The number of LVAD placements in ESRD patients has increased from 2010 to 2014 but mainly in White men in this five-year study. Although in-hospital mortality in ESRD patients undergoing LVAD was high (54%), there was a decrease in mortality from 2010 to 2014 (69%-48%, P < 0.05). Pulmonary HTN, age > 60, and diabetes are independent risk factors in this subset of patients for in-patient mortality.
12	Ullah (2020) [14]	HD in LVAD patients led to an increase in CO after UF despite a decrease in the mean blood pressure doppler (MDP) (mmHg) and increased heart rate.
13	Walther (2018) [15]	ESRD was found to be initially associated with a higher risk for in-hospital mortality after implantable LVAD placement, with >30% of persons dying before discharge. The risk is decreased in recent years in those with and without ESRD and becoming almost similar perhaps due to improved technology and experience. The ESRD group has similar in-hospital mortality as the non-ESRD group. Female sex and implantation in the setting of another cardiotomy were independently associated with higher mortality.

**Table 2:** Baseline study characteristics

Number	First author	Study type	LVAD	Other assist device	Number of patients	ESRD before LVAD	ESRD after LVAD	Laboratory/imaging		Heart or kidney transplantation
								Creatinine	Echo	
1	Agarwal (2019) [5]	Case report	Yes	N/A	1	Yes (cardiorenal syndrome)	Yes	N/A	N/A	N/A
2	Bansal (2018) [3]	Prospective cohort study	Yes	N/A	461	155 patients	Yes	N/A	N/A	Nine (5.8%) with ESRD received heart transplantation compared to 56 (25%) without ESRD; four cohort patients alive with heart transplant one year after LVAD placement compared to 29 patients with ESRD (13%)
3	Bavishi (2018) [6]	Retrospective study	-	Percutaneous mechanical circulatory support (pMCS)-assisted PCI/IABP	29,247	935 patients (3.2%)	N/A	N/A	N/A	N/A
4	Demirozu (2011) [7]	Prospective cohort study	Yes	N/A	107	15 patients required RRT prior to LVAD	Five patients	Cr clearance prior 64 ± 39 mL/minute improved to 92 ± 55 mL/minute (P = 0.041) after two months of LVAD support	N/A	N/A
5	El Sayed Ahmed (2014) [8]	Case report	Biventricular device (BiVAD)	ECMO days + total artificial heart (TAH)	1	Yes	Yes	Transplanted kidney: Cr, 1.3 mg/dL	TTE, 25% to 55%-60% posttransplant	Simultaneous heart and kidney transplantation 107 days after TAH implantation
6	Franz (2020) [9]	Case series	Yes	N/A	11	No (six patients with CKD)	Yes	Maintenance dialysis	N/A	One patient underwent a combined heart and kidney transplantation
7	Gilligan (2020) [10]	Case report	Yes	N/A	1	Yes	Switched to PD	N/A	N/A	HeartMate II LVAD as destination therapy
8	Hankinson (2019) [11]	Case report	Yes	N/A	1	CKD progressed to ESRD	Yes (six months post-implantation)	N/A	N/A	N/A
9	Ignaszewski (2017) [12]	Case report	Yes	N/A	1	Yes	Yes	N/A	N/A	N/A
10	Shah (2017) [13]	Retrospective cohort study	Yes	N/A	415	Yes	Yes	N/A	N/A	N/A
11	Ullah (2020) [14]	Case report	Yes	N/A	1	Yes	Yes	N/A	N/A	LVAD for destination therapy
12	Walther (2018) [15]	Retrospective observational cohort study	Yes	N/A	24,334	124 patients	Yes	N/A	N/A	N/A
13.	Walther (2018) [4]	Retrospective	Yes	N/A	96	74 on HD and 10 on PD	Yes	N/A	N/A	N/A

Discussion

In the United States, around 6.2 million adults have heart failure according to the Centers for Disease Control and Prevention [16]. Heart failure is a complex clinical syndrome that results from any functional or structural disorder that impairs ventricular filling or ejection of blood to the systematic circulation [17]. Guideline-directed medical therapy has helped improve symptoms and survival in patients with heart failure with reduced ejection fraction; however, many continue to reach stage D heart failure (HF), which requires other modified interventions [18]. The primary indication for cardiac transplantation is a patient with advanced heart failure refractory to optimal medical therapy. Ventricular assist devices (VADs) have become more common and are widely used as a bridge to therapy for patients to cardiac transplantation or as destination therapy in patients with advanced heart failure who are not suitable for transplantation. Mechanical circulatory support (MCS), although a viable strategy, also has complications. MCS-related complications such as infection, stroke, pump thrombosis, device failure, and suboptimal long-term survival remain challenges [19]. According to the Randomized Evaluation of Mechanical Assistance for the Treatment of Congestive Heart Failure (REMATCH) trial, LVAD implantation as a destination therapy improved survival compared with medical therapy alone [3].

About 50% of patients will develop chronic kidney disease within five years after heart transplantation, and 6% will require dialysis within 10 years [19]. Immunosuppressive therapy posttransplant with calcineurin inhibitors (CNIs) was found to cause acute and chronic intrinsic nephrotoxicity [20]. Different strategies have been implemented to reduce the doses of CNIs and even avoid their use completely to prevent renal dysfunction [20,21]. MCS is a risk factor for CKD development in these patients among other risk factors such as older age, female sex, lower glomerular filtration rate (GFR), and pretransplant inotrope use [20]. Before considering LVADs in this patient population, a multidisciplinary approach with a nephrologist should be considered in patients with GFR below 30 mL/minute/1.73 m^2^ or who are found to have significant proteinuria (>500 mg/d) [22,23]. The prevalence of heart failure is approximately 40% among patients with end-stage renal disease (ESRD), and 37% of patients die from heart failure [3]. Despite the high prevalence of heart failure in patients with ESRD, there is a lack of information regarding the use of LVAD in these patients as either a DT or BTT as few studies have evaluated outcomes among LVAD recipients with ESRD. Additionally, it was found that patients with ESRD at the time of LVAD placement had a poor prognosis, with most surviving less than three weeks [3].

MCS/LVAD Selection Criteria and Contraindications

Advanced heart failure patients should be referred to a center for evaluation and risk assessment to determine which patients would benefit from a heart transplant. The American College of Cardiology and the American Heart Association recommend common indications and contraindications for cardiac transplants (Tables 3, 4). Patient selection and timing are essential for LVAD therapy. Although preoperative risk assessment scores are imperfect, they have highlighted several risk factors, such as preexisting renal dysfunction, liver dysfunction, poor nutritional status, and coagulopathy, which have adverse effects on patient outcomes post-VAD implantation [23]. Patients were assigned a candidate transplant status based on the severity of illness, geographic distance between donor and recipient, length of time on the waitlist, and blood group compatibility [24,25].

**Table 3:** Indications for heart transplantation [25-26]

Indications for heart transplantation
Cardiogenic shock requiring either intravenous inotropic support (dobutamine, milrinone, etc.) or refractory cardiogenic shock requiring MCS
Persistent NYHA class IV congestive HF symptoms refractory to maximal medical therapy or resynchronization therapy
LVEF < 20%, peak VO_2_ < 12 mL/kg/minute
Refractory or intractable severe angina not amenable to medical or surgical therapeutic options
Recurrent life-threatening arrhythmias unresponsive to anti-arrhythmic therapy, catheter ablation, and/or implantation of an intracardiac defibrillator

**Table 4:** Contraindications for heart transplantation [25-26]

Absolute contraindications	Relative contraindications
Irreversible liver disease	Age > 70-72 years
Severe obstructive lung disease (FEV_1_ <1 L/minute)	Any active infection (with exception of device-related infection in VAD patients)
Active or recent solid organ or blood malignancy within five years	Severe obesity (BMI > 35 kg/m^2^) or cachexia
Irreversible pulmonary artery hypertension (pulmonary arterial systolic pressure (PASP) > 60 mmHg), pulmonary vascular resistance (PVR) > 5 wood units despite the use of vasodilators	Diabetes mellitus with end-organ damage
Severe irreversible systemic illness	Irreversible renal dysfunction (GFR < 30 mL/minute/1.73 m^2^)
Clinically severe symptomatic cerebrovascular disease	Severe cerebrovascular disease or peripheral vascular disease
	Heparin-induced thrombocytopenia within 100 days
	Acute pulmonary embolism within 6-8 weeks
	Psychosocial instability or lack of social support or resources to permit ongoing therapy
	Drug, tobacco, or alcohol abuse within six months
	Inability to comply with medication therapy on multiple occasions

Pathophysiology of Renal Dysfunction in LVAD

Renal dysfunction has been identified as a risk factor for adverse outcomes in heart failure patients requiring LVAD support [1]. The relationship between renal disease and poor prognosis after LVAD implantation has not been completely understood and remains multifactorial. In patients with advanced heart failure, renal dysfunction was thought to be secondary to a cardiorenal syndrome, preexisting renal disease, or a combination of both [26,27]. Renal dysfunction may also be due to intrinsic disease from long-standing comorbidities or fluctuating acute and chronic hemodynamic changes causing venous congestion and poor renal perfusion [28]. Cardiorenal syndrome is a complex syndrome commonly seen in patients with advanced heart failure due to decreased renal perfusion. The renal parenchymal disease worsens over time with repeated AKI events, oxidative stress, and inflammation, resulting in an eventual progression to intrinsic CKD [28].

Several studies have examined the association of right ventricular (RV) failure with adverse outcomes following LVAD implantation. A patient with renal failure is more likely to suffer from right ventricular failure [1]. As RV failure develops, the dysfunctional right ventricle is unable to provide the left ventricle with adequate preload, resulting in a subsequent cascade of adverse hemodynamic events consisting of a progressive decrease in LV cavity size, decreased LVAD flow, and decreased systemic and renal blood flow [28]. One limitation to studying the pathophysiology of renal dysfunction in LVAD patients is that LVAD implantation trends have changed from historically pulsatile to exclusively continuous flow devices. Continuous flow left ventricular assist devices (CF-LVADs) result in increased diastolic pressures and continuous flow physiology. In conjunction with RV failure and venous congestion, an increased diastolic pressure could play a crucial role in developing kidney dysfunction [28].

ESRD and Hemodialysis (HD) Versus Continuous Renal Replacement Therapy (CRRT) in LVAD Patients

Patients with ESRD represent a unique patient population to undergo LVAD implantation since they are already receiving one form of chronic life support [3]. A study conducted by Bansal et al. focused on patients with ESRD (median duration of ESRD of four years) at the time of LVAD placement, which included about 85 patients who were receiving dialysis at the time of LVAD placement [3]. Only half of the patients in this study with ESRD survived at the time of LVAD placement. The median survival was 16 days after LVAD placement, with high in-hospital mortality, suggesting that patients were being offered these procedures when patients were either actively dying or were near death. Despite having ESRD, some patients survived longer and underwent heart transplants, suggesting that LVADs may be beneficial for some patients with ESRD. However, there is a need for additional studies, particularly given the current advances in LVADs and the diverse ESRD populations.

A retrospective analysis of 4,917 patients from the Interagency Registry for Mechanically Assisted Circulatory Support (INTERMACS) who had CF-LVAD implantation revealed that patients with estimated glomerular filtration rate (eGFR) < 30 mL/minute/1.73 m^2^ or those with dialysis dependence had excess mortality of 22% in the first three months [29]. An additional study demonstrated that patients with a preoperative GFR < 15 mL/minute/1.73 m^2^ who were prophylactically initiated on temporary renal replacement therapy postoperatively showed an increase in GFR to >30 mL/minute/1.73 m^2^ in about 11 of the 21 patients studied [30]. Among 17 patients in a study by Lamba et al. who required chronic intermittent hemodialysis (n = 13) or peritoneal hemodialysis (n = 4) prior to implantation, 14 patients required CRRT prior to implantation [31]. Ten patients of the 17 patients on chronic intermittent HD underwent CF-LVAD as BTT. Among the 14 patients on CRRT, eight underwent CF-LVAD as BTT and six as DT. The study results showed that patients on chronic intermittent dialysis had acceptable survival after CF-LVAD implantation, while those on CRRT prior to CF-LVAD had poorer outcomes. CF-LVADs are less preload-dependent than the earlier pulsatile flow devices, perhaps making volume shifts with ultrafiltration in hemodialysis less concerning [15].

Outcomes With LVAD in End-Stage Renal Disease

The number of LVAD implantations in patients with ESRD has risen over time. According to one study, this increased from 3.3% in 2010 to 5.2% in 2014 (P trend < 005) [13]. In one study, the results from a 762-day follow-up period showed that 81.9% of LVAD patients with ESRD died as compared to 36.4% of LVAD patients without ESRD. The median survival time for patients with ESRD was believed to be less than three weeks [13]. Another study reported in-hospital mortality after LVAD of 35.6% (95% CI, 17.3%-54.0%) in patients with ESRD compared to 14.7% in non-ESRD patients (95% CI, 13.4%-15.9%) [15]. A decline in mortality has been documented among this patient population. The one-year cumulative mortality rate in 2012-2014 remained high at 51.5% compared to 63.4% in 2006-2009 [15]. In another study, the cumulative incidence mortality after one year decreased from 64.5% in 2006-2009 to 55.6% in 2010-2011 and 42.5% in 2014-2021 (P < 0.05) [20]. ESRD patients with pulmonary hypertension, older age (age > 60), and diabetes are independently associated with a poor prognosis following LVAD implantation [13]. Female sex, other cardiac surgeries, and early placement of LVADs are risk factors with higher mortality in LVAD recipients, regardless of having ESRD [5]. ESRD alone increased the risk of readmission within 30 days [7]. A study evaluated the use of extracorporeal life support (ECLS) or biventricular device (BiVAD) in patients in cardiogenic shock bridged to heart transplantation and found that patients with extracorporeal support had higher rates of renal failure (P = 0.02) compared to the BiVAD patients [32].

The study of Bansal et al. had several limitations that raised concern that patients with ESRD underwent LVAD implantation too late [3]. Furthermore, this study relied primarily on diagnostic and procedure codes in administrative data to define and characterize the cohort but provided no clinical context or indication as to why and what type of LVADs were placed. Factors associated with the increased mortality seen were not discussed if directly related to a patient being ESRD on HD rather than a complication of LVAD itself such as infection or bleeding or simply patients with worsening advanced heart failure. In the study of Bavishi et al., they examined the underlying causes of readmission for many patients with MCS [6]. CHF (systolic or diastolic) ranked highest among the causes of readmission at 22%, followed by stable CAD (11%). This cohort consists mostly of patients hospitalized for acute myocardial infarction with concomitant HF or cardiogenic shock. The other limitations of the study included overestimating the readmission rate and an inability to determine whether readmissions were staged for subsequent PCI. It is unclear from this study if ESRD affected readmission. In a case series, it has been shown that outpatient hemodialysis centers and LVAD treatment teams can collaborate to provide hemodialysis to LVAD-dependent patients; however, more studies are required [9].

## Conclusions

Limited data and literature are available on the use of LVADs in patients with end-stage renal disease, and studies are limited to small case series and case reports and a few retrospective studies. This has limited the use of mechanical circulatory support for patients with advanced heart failure with ESRD. It is not clear from the studies described whether deaths were directly related to ESRD or just complications associated with LVAD placement or associated complications. The adverse effects of LVAD implantation, such as infections, bleeding, strokes, renal failure, and right heart failure, are known to result in poor outcomes. LVADs are found to have an acceptable survival for carefully selected patients on dialysis. Even so, this still requires multidisciplinary approaches and shared decision-making. As of today, there have been limited studies regarding which hemodynamic strategy is most effective in this patient population and if such an approach may improve survival rates with the current advances in left ventricular assist devices.
